# Pompe Disease: a Clinical, Diagnostic, and Therapeutic Overview

**DOI:** 10.1007/s11940-022-00736-1

**Published:** 2022-08-04

**Authors:** David Stevens, Shadi Milani-Nejad, Tahseen Mozaffar

**Affiliations:** 1 Departments of Neurology, 200 S. Manchester Avenue, Ste. 206, Orange, CA 92868, USA; 2 Pathology & Laboratory Medicine, School of Medicine, University of California, Irvine, USA; 3 The Institute for Immunology, School of Medicine, University of California, Irvine, USA

**Keywords:** Pompe disease, Glycogen storage disease II (GSDII), Acid maltase deficiency, Alpha-glucosidase deficiency, Enzyme replacement therapy, Gene therapy

## Abstract

**Purpose of Review:**

This review summarizes the clinical presentation and provides an update on the current strategies for diagnosis of Pompe disease. We will review the available treatment options. We examine newly approved treatments as well as upcoming therapies in this condition. We also provide commentary on the unmet needs in clinical management and research for this disease.

**Recent Findings:**

In March 2015, Pompe disease was added to the Recommended Uniform Screening Panel (RUSP) and since then a number of states have added Pompe disease to their slate of diseases for their Newborn Screening (NBS) program. Data emerging from these programs is revising our knowledge of incidence of Pompe disease. In 2021, two randomized controlled trials involving new forms of enzyme replacement therapy (ERT) were completed and one new product is already FDA-approved and on the market, whereas the other product will come up for FDA review in the fall. Neither of the new ERT were shown to be superior to the standard of care product, *alglucosidase*. The long-term effectiveness of these newer forms of ERT is unclear. Newer versions of the ERT are in development in addition to multiple different strategies of gene therapy to deliver GAA, the gene responsible for producing acid alpha-glucosidase, the defective protein in Pompe Disease. Glycogen substrate reduction is also in development in Pompe disease and other glycogen storage disorders.

**Summary:**

There are significant unmet needs as it relates to clinical care and therapeutics in Pompe disease as well as in research. The currently available treatments lose effectiveness over the long run and do not have penetration into neuronal tissues and inconsistent penetration in certain muscles. More definitive gene therapy and enzyme replacement strategies are currently in development and testing.

## Introduction

Pompe disease, also known as glycogen storage disease type II (GSD II) or acid maltase deficiency (AMD), is a genetic disorder caused by a deficiency of the acid alpha-glucosidase (GAA) enzyme, due to recessive mutations in the GAA gene, which leads to accumulation of lysosomal glycogen [1], diffusely but primarily affecting the skeletal and cardiac muscle tissue. More than 300 different mutations have been described in the GAA gene. The clinical presentation of Pompe disease is a spectrum between the cardiac and skeletal muscle dysfunction and has wide variability. It is divided into two forms which are referred to as infantile onset Pompe disease (IOPD), described by Johannes Pompe in the 1930s and late-onset Pompe disease (LOPD), described by Andrew Engel in 1969 [2, 3]. IOPD has more severe cardiac involvement with hypertrophic cardiomyopathy, hypotonia, and respiratory insufficiency and is often fatal within 1 year of age without treatment. LOPD presents primarily as a muscle disease and has a more insidious course. LOPD can have onset anywhere from infancy to late adulthood. It presents with a pattern of symmetric limb-girdle muscle weakness and LOPD was till recently classified additional under the limb-girdle muscular dystrophy umbrella, with a designation of LGMD2V [4]. However, the revised classification system removed Pompe disease [5•]. It is the severity of the enzyme deficiency that determines which phenotype (IOPD vs. LOPD) will result. Patients with IOPD have a severe or complete GAA deficiency with < 1% residual enzyme activity, whereas LOPD is caused by only a partial deficiency (< 30% residual activity) of GAA [6].

## Epidemiology

The traditional estimate of incidence is around 1/40,000 overall [7, 8], with about 3/4 of the cases as LOPD and 1/4 as IOPD. Incidence can vary widely among different ethnic groups and has historically been based on retrospective data from carrier frequencies. The populations that appear to be at higher risk include people of African American, Taiwanese, Dutch, and Israeli descent. However, now that newborn screening protocols are being put in place, we are getting more definitive incidence frequencies.

Newborn screening (NBS) from California showed a birth prevalence of 1/25,200 [9•]. NBS in Illinois, Pennsylvania, and Missouri have shown incidences of 1/23,596, 1/16,095, and 1/10,152 respectively [10–12]. Analysis of NBS data in Japan showed an overall incidence of ~ 1/37,000 from 2013 to 2020 [13]. Studies from Taiwan have shown birth prevalence rates of 1/26,466 or 1/20,114 for LOPD and 1/67,047 for IOPD [14•, 15]. Overall, the incidences from these newer studies for Pompe disease are higher than the previously estimated 1/40,000 as above. For IOPD specifically, the data has been quite variable. The screening data from Japan, California, Pennsylvania, and Illinois has shown IOPD incidence ranging from around 1/200,000 to 1/300,000 [9•, 11–13]. However, the incidence rates seen in Taiwan and Missouri are much higher at 1/67,047 and 1/46,700 respectively [12, 15]. Further data collected with newborn screening in different parts of the world will be key in gaining a better understanding of the epidemiology of this disease.

The new data does raise an interesting conundrum. If the incidence is indeed so much higher, it approximates the incidence of relatively more common neuromuscular disorders such as facioscapulohumeral muscular dystrophy (FSHD) (1 in 15,000) [16] and myotonic dystrophy (DM1) (1 in 8000) [17]. The prevalence of Pompe disease in the Neuromuscular Clinics or the Muscular Dystrophy Association (MDA) clinics is nowhere close to those of FSHD or DM1, which begs the question whether these patients with Pompe disease are misdiagnosed as other musculoskeletal disorders or whether not all mutations have the same penetrance and may not manifest disease?

## Pathophysiology

Acid alpha-glucosidase (GAA) facilitates the breakdown of glycogen to glucose within the lysosomes of cells throughout various tissues in the body [6]. With a deficiency of this enzyme, as seen in Pompe disease, there is abnormal accumulation of glycogen and progressive expansion of these glycogen-filled lysosomes. The skeletal and cardiac muscle are the most affected. Primary mechanisms of cellular injury are lysosomal rupture and autophagy. In 1970, Engel reported that autophagic function was abnormal in Pompe patients [3], and more recent mouse models showing accumulation of autophagosomes have supported this idea [6]. Typically, autophagy works in nutrient poor states by recycling intracellular material to supply amino acids for energy production. Additionally, autophagy helps clear out mis-folded proteins and other intracellular debris [6]. This process entails autophagosomes forming and collecting intracellular contents, after which they fuse with lysosomes to degrade the collected material. It is thought that this autophagic buildup may work in conjunction with the expanded lysosomes from glycogen build up, and their rupture spilling the contents into the sarcoplasm, to cause dysfunction and injury to muscle [6].

## Diagnostic evaluation

The diagnosis of Pompe disease is ultimately confirmed with enzyme assays and genetic testing. Clinical history, exam, muscle enzymes, and electromyography (EMG) are the core of the initial workup and are used to help determine which patient should undergo further testing specific to Pompe disease, particularly in the setting of LOPD [1].

A clinical history of a limb-girdle pattern of weakness that is slowly progressive over years is the typical clinical phenotype of LOPD. Respiratory insufficiency is present in the majority of patients [18]. Examination will show a limb-girdle pattern of weakness with most prominent weakness typically affecting thigh adductors [18]. This is often accompanied by postural changes such as lumbar lordosis or camptocormia, with scapular winging. Laboratory workup is expected to show an elevated creatine kinase (CK) level in the majority of patients but can be normal. The CK level will typically not be higher than 2000 U/L, and average around 600–700 U/L [19]. Electromyography will classically show an irritable myopathy affecting the proximal muscles. One key feature often seen in Pompe disease on EMG is myotonic discharges in the paraspinals muscles. The IPANEMA study found that within patients with proximal muscle weakness and elevated CK levels presenting undiagnosed to academic neuromuscular centers, the prevalence of LOPD was 1% [20••].

More definitive and specific testing for Pompe disease consists of muscle biopsy, enzyme assays, and genetic testing. Muscle biopsy shows vacuolated fibers filled with glycogen as can be seen on PAS or acid phosphatase staining [21]. Enzyme assays are able to detect a deficiency of acid alpha-glucosidase, as seen in this condition. This can be done on either muscle tissue or blood spot, leukocytes, or fibroblasts [22•, 23]. Some would consider enzyme deficiency confirmed on two different sample types to be diagnostic even without muscle biopsy or genetic testing. Lastly, genetic testing with sequencing of the GAA gene to detect mutations is another definitive diagnostic step [23]. As mentioned above, over 300 different mutations have been identified in the GAA gene. Specific mutations vary between IOPD and LOPD and across different ethnic groups.

It is common for there to be a 12–13-year delay in diagnosis from onset of symptoms for LOPD due to the rarity of the condition and insidious progression. However, IOPD is diagnosed rapidly with the assistance of newborn screening panels. In 2015, Pompe disease was added to the Recommended Uniform Screening Panel (RUSP), and as of January 2021 there were 23 states screening for Pompe disease. The common method used for newborn screening is to start with a blood spot enzyme assay, followed up by genetic sequencing for confirmation if enzyme levels are reduced. One challenge is that the enzyme assays used cannot reliably differentiate between IOPD and LOPD [15]. Therefore, confirmatory testing with genetic sequencing or other workup (CK, cardiac evaluation) can help determine if a positive blood spot result indicates already symptomatic IOPD necessitating early treatment, or LOPD which may not manifest until years or decades later. If the clinical picture fits IOPD and the assay shows deficiency, treatment will often begin prior to genetic confirmation, which can take weeks to result.

## Diagnostic challenges

A challenging situation arises when LOPD is diagnosed genetically at birth with newborn screening panels before any symptoms are present. This occurs often because LOPD is much more common than IOPD and LOPD accounts for 75% of all cases of Pompe disease diagnosed through NBS. At this time, there is no clear consensus on how to manage these patients and the guidelines, developed primarily by a group of metabolic geneticists without much neurology input [24], are not uniformly enforced. Many of these kids will not manifest any symptoms until their teenage years or much later. It is not known if early treatment can have any prophylactic effect to delay or prevent symptoms, or if we should wait until symptom onset or laboratory abnormalities arise to initiate treatment. Further, insurance companies generally do not reimburse for asymptomatic checks or care, so it is not clear who is supposed to pay for these routine surveillance visits. There is a desperate need for sensitive biomarkers. In addition to serum CK levels and urinary excretion of tetrasaccharides (Hex4), an interesting potential biomarker is MR spectroscopy that can assess glycogen levels in tissue, and has been demonstrated to be useful in quantifying hepatic and muscle glycogen in glycogen storage diseases and other metabolic conditions [25–27]. This or other advanced imaging techniques could serve as non-invasive methods of early disease detection in these asymptomatic LOPD cases to determine when to initiate treatment.

The knowledge of the diagnosis, in individuals without symptoms or functional loss, may cause anxiety or depression and unnecessary modifications to their lifestyles as well as unnecessary treatments. This is an area of Pompe disease that needs much more study. The optimal time to initiate ERT is not settled and adds to the complexity of managing pre-symptomatic or asymptomatic patients.

Another major challenge relates to diagnosis of Pompe disease is GAA pseudodeficiency. There are haplotypes of the GAA gene that cause GAA pseudodeficiency, which shows up as low enzyme activity on assays but does not have any clinical effects or lead to symptomatic disease. Using enzyme assays in isolation can lead to many false-positives in the initial screening steps. Therefore, it is important to follow up with genetic testing in these cases, to detect the pseudodeficiency haplotypes, which presents often as homozygous for the c.[1726A; 2065A] pseudodeficiency allele [28]. This genotype appears to be more common than Pompe disease. In Illinois, the birth prevalence of pseudodeficiency was 1/17,546, in Missouri it was 1/8811, in California it was 1/22,658, and in Pennsylvania it was 1/35,409 [9•, 10–12]. Other countries have shown even higher rates of pseudodeficiency such as 1/1368 in Taiwan and 1/8747 in Japan [13, 15]. The IPANEMA study showed a prevalence of 1% for both LOPD and pseudodeficiency alleles in that population [20••].

## Treatment

### Supportive care/complications

In Pompe disease, in addition to the disease-modifying treatments, overall management of this condition requires symptomatic management and screening for complications, particularly in LOPD, preferably through a multidisciplinary clinic allowing a team of allied health care professionals, including physical therapy, speech therapy, respiratory therapy, dieticians, and genetic counselors, ensuring that all aspects of patient care are being addressed. Additionally, coordination and communication between different medical teams such as neurology, genetics, pulmonology, gastroenterology, and cardiology can be crucial for proper management.

Light exercise or aerobic exercise such as swimming is very beneficial in maintaining mobility and functionality. The goal is to stay active and exercise as able without inducing muscle soreness or prolonged recovery times after activity.

The two most life-threatening complications of LOPD are related to respiratory and cardiac dysfunction. Patients should undergo routine pulmonary function testing to determine their degree of respiratory insufficiency related to diaphragm weakness. When a patient’s forced vital capacity (FVC) approximates 50% predicted, it is advised to initiate non-invasive ventilation (NIV) with a positive airway pressure respiratory assist devices that help support the diaphragm function. Without this supportive treatment, patients continue to have sleep disordered breathing and may begin to retain CO_2_ chronically, leading to headaches, daytime sleepiness, and lack of energy. There should be a low threshold to order polysomnographic studies to monitor for it. It has been shown that NIV can improve survival and quality of life [29]. Cardiac management is crucial in IOPD, but cardiac dysfunction is less common in LOPD [30]. It is important to screen patients regularly for cardiac hypertrophy or conduction abnormalities.

An understudied area in the management of LOPD is in regard to vascular malformations, which appear to be quite common in this disease, with 60% of LOPD patients showing intracranial arterial abnormalities, such as vertebrobasilar dolichoectasia and unruptured aneurysms [31, 32]. These types of malformations could place patients at risk for strokes, compression, or hemorrhage in severe cases. However, there are no guidelines regarding monitoring for these complications, as well as other complications, in Pompe disease [33].

### Disease-modifying therapies

The treatment of Pompe disease has mainly been targeted at correction of the underlying GAA deficiency. This has included trying to supplement the enzyme in various ways, and gene therapy allowing endogenous production of the GAA enzyme.

### Currently available treatments

Enzyme replacement therapy (ERT) is given as human recombinant GAA (rhGAA) has been used in Pompe disease as early as the 1970s primarily studied initially for IOPD [34, 35]. A randomized controlled trial in 2006 for IOPD ultimately led to FDA approval of rhGAA [36] for all forms of Pompe disease. The study in 2006 was done in IOPD and showed that ERT improved overall survival and ventilator-free survival in patients [37], and suggested earlier intervention provided greater benefit. Significant reduction in left ventricular hypertrophy was seen in all surviving patients in this study as well. Despite these clear benefits seen in early life, long-term follow up of IOPD patients still shows significant morbidity and mortality and requires further study [38]. While ERT has allowed these patients to survive cardiac and respiratory effects into childhood and often achieve independent walking, many of them start to experience decline in skeletal muscle strength years later and develop cardiac arrhythmias even when cardiac hypertrophy has been avoided or reduced with ERT. Additionally, these IOPD that survive into childhood with ERT go on to develop other problems including hearing loss, speech dysfunction, cognitive impairment, and GI as well as respiratory dysfunction [39]. There is an unmet need for management of these patients.

With the publication of the LOTS data in 2010, enzyme replacement therapy with alglucosidase alfa in LOPD was shown to improve or at least slow the decline of ambulation, arm and leg function, and respiratory function [39]. However, the effectiveness seems to wear off after 2–3 years and patients return to their slow decline [40•, 41]. Because of this lack of a sustained response to alglucosidase alfa, more recently two new forms of enzyme replacement therapy were developed and tested in clinical trials to meet the unmet need in Pompe Disease. These new iterations of ERT, avalglucosidase alfa and cipaglucosidase alfa plus miglustat, were compared to alglucosidase alfa (the standard of care) in the COMET and PROPEL trials, both published in December 2021 [42••, 43••]. Avalglucosidase alfa (COMET trial) is a form of rhGAA that is designed with enhanced targeting of mannose-6-phosphate receptors, through chemical conjugation of synthetic linkers, to increase the uptake of rhGAA into cells on the target tissues [42••]. The PROPEL trial examined a two-component therapy that included cipaglucosidase alfa, an rhGAA with enhanced glycosylation for improved cellular uptake, through clonal selection of rhGAA with CHO-cell derived M6P and bis-M6P moieties, and miglustat, a stabilizer of the cipaglucosidase alfa molecule, which prolongs half-life and increases distribution [43••]. Both new forms of ERT were shown to be non-inferior to alglucosidase alfa and did not meet the prespecified criteria for superiority compared to alglucosidase alfa [42••, 43••]. At this point, we do not have the long term data on these new agents to see how they will fare after 2–3 years of treatment (Table 1).

## Treatments under investigation

**Fig. 1:**
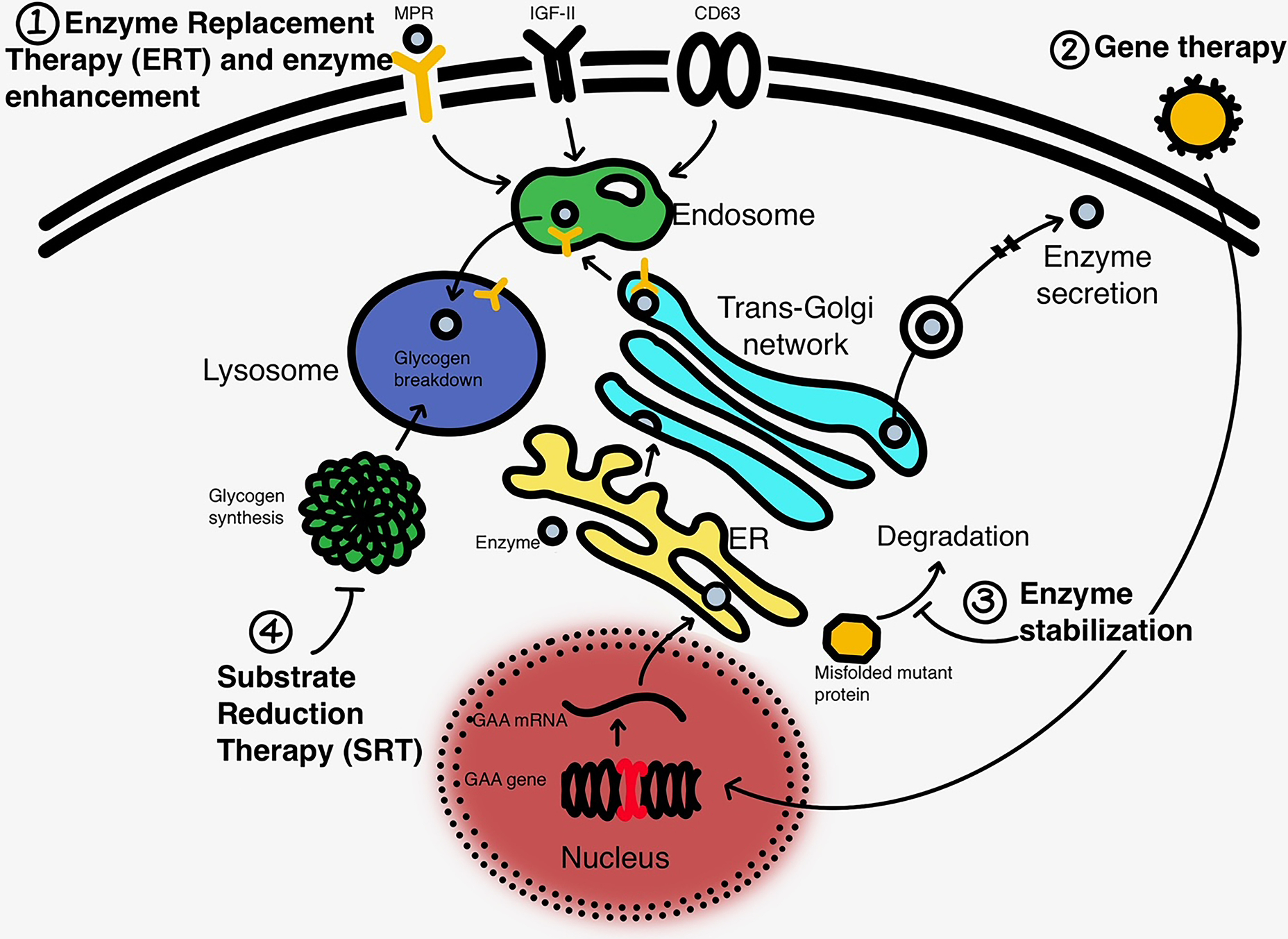
A schematic for the mechanism of action within the cell (liver or skeletal muscle) for the current and upcoming therapies in Pompe Disease. (1) Enzyme replacement treatments with enhancement in the enzyme through modifications that allow better delivery into muscle either through the mannose-6-phosphate receptors (MPR), IGF-II receptor or binding through a monoclonal antibody to CD63 conjugated with the enzyme; (2) delivery of full GAA gene through gene delivery using a viral (AAV or Lentiviral) vector, with either liver or muscle targeting; (3) enzyme stabilization to allow more of the enzyme to remain intact, and the catalytic activity protected from degradation; and (4) substrate reduction strategy, primarily targeted to glycogen synthetase using either small molecule or genetic manipulation.

Newer treatments under development or under investigation are depicted in Table 2 and shown graphically in Fig. 1.

### Enhancements in enzyme replacement strategies

Alternate enzyme replacement strategies have been tried or being developed. A recent trial of a new glycosylation-independent lysosomal targeting (GILT)-tagged ERT that utilized the IGF-II receptors in skeletal muscles to allow entry of the enzyme into the muscles was undertaken but was terminated early due to development of significant symptomatic hypoglycemia [33]. Newer approaches include a combination of gene therapy to target the liver and using monoclonal antibody (to CD63 or ITGA7)-conjugates with the enzyme to allow for targeted entry into skeletal muscles [44•]. Another effort to show improvement in ERT delivery through an antibody-enzyme fusion product showed safety and tolerability, but the program was discontinued due to lack of funding [45•].

### Gene therapy

Gene therapy is a very exciting treatment modality on the horizon for Pompe disease. Through a one-time treatment of the transgene that would then endogenously produce the enzyme, this would obviate the need for chronic ERT therapy biweekly. While majority of the gene therapies use adeno-associated virus vectors (AAV) [46] for a one-time delivery of a non-integrating vector carrying the transgene, one group is proposing use of lentivirus-driven correction of autologous hematopoietic stems cells and reinfusion of cells. The gene therapy approaches differ as well with trials using a liver-directed approach vs. a muscle-directed approach.

For liver-directed therapy, treatment would consist of a one-time intravenous (IV) infusion of the AAV-packaged transgene, which would be delivered into the nucleus of liver cells and would begin to produce the therapeutic protein; in this case GAA, in a sustainable fashion. This would create liver depot for GAA production and the secretable GAA released into the bloodstream and available for delivery to skeletal and cardiac muscle tissues. This approach takes advantage of the high tropism of AAV vectors for hepatic cells, requiring lower vector dose. Further, proteins produced in the liver appear to be immunologically privileged. This form of gene therapy is currently under investigation in phase 1/2a trials (NCT04093349 and NCT03533673). There have been no major safety signals and preclinical findings have shown promising results of reduced glycogen accumulation in skeletal and cardiac tissue and improvement of muscle function [47, 48]. However, with all the AAV approaches, there still remain safety concerns related to capsid-related hepatotoxicity as well as development of neutralizing antibodies to the capsid. Currently, individuals who have pre-existing antibodies to AAV are excluded from participation due to concerns for premature neutralization of the capsid and the transgene.

The muscle approach is another exciting opportunity, either intravenous approach with muscle targeting or direct intramuscular approach. With either path, the GAA transgene would be delivered to muscle cells, and begin to produce a functional GAA enzyme to mitigate lysosomal glycogen accumulation in those cells. An ongoing trial uses a skeletal muscle targeting approach, given as an IV infusion using AAV vector with muscle-specific serotype and promoters (NCT04174105). As only 1% of enzyme produced in the liver actually makes it into the target organs (skeletal and cardiac muscle), muscle-directed AAV therapy resolves this problem. The IV delivery method of muscle-directed therapy would aim to deliver the AAV vector systemically to all muscle tissue, but would require a much higher vector dose, which may increase the likelihood of anti-GAA antibodies developing and interfering with the therapeutic effect as well as hepatotoxicity and cardiomyopathy. Preclinical data for muscle-directed IV therapy has been positive in showing substantial clearance of lysosomal glycogen in skeletal and cardiac muscle tissue in mice [49]. Another approach consists of direct intramuscular (IM) injection to deliver the vector directly to muscle fibers. One advantage of the IM therapy is that certain muscles that are more affected could be targeted, such as the diaphragm, allowing for more flexibility and specificity of treatment. However, this is also a disadvantage in that the positive effects appear to be quite local at the site of injection, which may suggest the need for multiple injections at different sites and potentially a higher risk for antibody development. Preclinical data for this muscle-directed IM therapy has been encouraging in showing success of intralingual and intradiaphragmatic injection in mice [50, 51•]. Overall gene therapy treatment options are a very exciting area of on-going research and show great promise for more definitive long-term treatment of Pompe disease.

Another approach being considered is an intrathecal or intraventricular approach to maximize delivery into the CNS, especially for IOPD cases, where the burden of disease, in addition to the cardiac muscles, is maximal in motor neuron cells.

Finally, an antisense approach to improve the IVS splicing in Pompe disease was discussed by the Erasmus group at an international meeting (Nadine van der Beek—personal communication). This offers a promising approach to improve enzyme production through mitigation of the most common genetic abnormality in Caucasian patients with Pompe disease.

### Glycogen reduction strategies

Alternative strategy of substrate (glycogen) reduction is being studied with the aim of reducing the amount of glycogen in cells, either through small molecules, currently in phase 1 in healthy individuals (NCT05249621), or through genetic approaches [52, 53, 54•], thus delaying the onset of symptoms from Pompe disease. This treatment can be used either alone or as an adjunct to the ERT. This approach would also be applicable for other glycogen storage disorders, and may be particularly attractive for delaying disease in at-risk asymptomatic individuals.

## Unmet needs

Unmet needs in clinical care as well as in research are described in Table 1. In addition to the need to improve diagnostic times and diagnosing these patients earlier, before the burden of disease becomes large, and the muscles accrue irreversible damage, there are research unmet needs related to inadequacies of the current available treatments. Additionally, the current outcome measures used in quantifying disease burden, monitoring disease progression, and to quantify treatment-related outcomes are woefully inadequately. Six-minute walk test, traditionally used in this disease unfortunately, is not sensitive enough especially in younger individuals and subject to training effects. Forced vital capacity does not change till much later in the disease and is not a direct measure of diaphragmatic strength. There is a desperate need to develop newer and more sensitive outcome measures and biomarkers in this disease. There has been considerable work that is being done to validate magnetic resonance imaging (MRI) as an outcome measure. There are new strategies to develop MR-spectroscopy as an outcome measure, since it has the potential to assess glycogen burden in muscles non-invasively.

## Conclusion

Pompe disease is a heterogeneous disorder with bimodal presentation. Enzyme replacement therapy is currently the mainstay of treatment for all forms of Pompe disease but the current therapies have significant unmet needs. ERT improves overall survival, ventilator free survival, and cardiac function in infantile cases, and stabilizes mobility and skeletal and respiratory muscle strengths in adult. However, after 2–3 years, ERT begins to lose effectiveness and patients continue to decline. Two new ERT treatments were showed to be non-inferior to the existing standard of care but could not establish superiority, and it is not clear if these treatments would lose effectiveness in a few years. There are significant unmet needs in terms of lack of guidance on management of LOPD patients diagnosed at birth, potentially long before the disease will manifest any symptoms. Similarly optimal outcome measures to measure clinical phenotype, progress, and treatment outcomes need to be defined. Newer promising treatments with liver-directed and muscle-directed gene therapies are in clinical trials, and these if they are effective, would result provide long-lasting therapy with only a single necessary treatment, creating endogenous production of the GAA enzyme to correct the underlying deficiency and cardiac and skeletal muscle pathology. Additional development include newer enhanced forms of ERT as well as substrate (glycogen) reduction strategies, which are about to enter clinical trials for LOPD. In addition to the current and upcoming therapies, there remains a need for a multidisciplinary approach and more wholistic approach to care of patients with Pompe disease.

## Figures and Tables

**Table 1:** Unresolved clinical and research questions in Pompe disease

• Prevent the current significant delays and diagnostic odysseys in diagnosis of Pompe Disease • Optimal time to start disease modifying therapy, especially in Late-Onset Pompe Disease • Delay disease onset by use of strategies such as substrate reduction • Reason for the disconnect between the high birth incidence rate of Pompe Disease and lower prevalence of Pompe cases in neuromuscular clinics • Make treatments more effective ○ Prolong the effectiveness of ERT ○ Better delivery to target organs (skeletal muscle, cardiac muscle, CNS) ○ Mitigate neutralizing antibodies to GAA ○ Mitigate anti-capsid antibodies • Develop better and more sensitive outcome measures for measuring disease progression and treatment outcomes

**Table 2:** Current treatment strategies in place or being considered in Pompe disease

• Enzyme replenishment through enzyme replacement therapy (ERT) and enzyme enhancement ○ BisM6P enrichment ▪ Through chemical conjugation of synthetic linkers ▪ Through clonal selection of rhGAA with CHO-cell derived M6P and bis-M6P moieties ○ IGF-2 receptor related GILT-tagging ○ Monoclonal antibody driven entry into muscle using CD63 and ITGA7 • Minimizing rhGAA inactivation in circulation with small molecule enzyme stabilizer • Delivery of full length gene through viral delivery for production of endogenous GAA ○ Liver approach ○ Muscle approach ○ Intrathecal or intraventricular approach • Ex-vivo genetic modification of hematopoietic (CD34 +) stem cells (HSCs) through a lentiviral approach to express GILT-tagged GAA enzyme • Antisense approach to improve the IVS splicing variant • Substrate Reduction therapy (SRT) ○ Small molecule approach ○ Antisense approach
